# Minicircle Delivery to the Neural Retina as a Gene Therapy Approach

**DOI:** 10.3390/ijms231911673

**Published:** 2022-10-02

**Authors:** Federica Staurenghi, Michelle E. McClements, Ahmed Salman, Robert E. MacLaren

**Affiliations:** 1 Nuffield Laboratory of Ophthalmology, Clinical Neurosciences, University of Oxford, Oxford OX3 9DU, UK; 2 Oxford University Hospital, Oxford OX3 9DU, UK

**Keywords:** minicircle, non-viral, gene therapy, retina

## Abstract

Non-viral gene therapy has the potential to overcome several shortcomings in viral vector-based therapeutics. Methods of in vivo plasmid delivery have developed over recent years to increase the efficiency of non-viral gene transfer, yet further improvements still need to be made to improve their translational capacity. Gene therapy advances for inherited retinal disease have been particularly prominent over the recent decade but overcoming physical and physiological barriers present in the eye remains a key obstacle in the field of non-viral ocular drug delivery. Minicircles are circular double-stranded DNA vectors that contain expression cassettes devoid of bacterial DNA, thereby limiting the risks of innate immune responses induced by such elements. To date, they have not been extensively used in pre-clinical studies yet remain a viable vector option for the treatment of inherited retinal disease. Here, we explore the potential of minicircle DNA delivery to the neural retina as a gene therapy approach. We consider the advantages of minicircles as gene therapy vectors as well as review the challenges involved in optimising their delivery to the neural retina.

## 1. Introduction

Gene therapy for inherited retinal disease continues to be at the forefront of new developments in translational medicine. In the previous decade, a wide array of pre-clinical and clinical trials led to rapid advancements in viral vector delivery. Currently, the primary vector for targeted therapy in the neural retina has proven to be the adeno-associated virus (AAV), which has now been used in various forms across many trials, exhibiting an encouraging safety and efficacy profile [1]. Whilst the therapeutic cargo is shifting from gene supplementation to gene editing elements, AAV remains the primary delivery mode for targeting cells in the neural retina. Non-viral approaches for retinal gene therapy have been reviewed in recent years [2,3,4,5], but minicircles are a less explored alternative that warrant specific consideration. Given that there is still an inability to reliably deliver larger therapeutic transgenes, pursuing minicircle delivery to the neural retina continues to be a worthwhile venture and may provide a solution to this ongoing problem.

## 2. Minicircles

**Figure 1:**
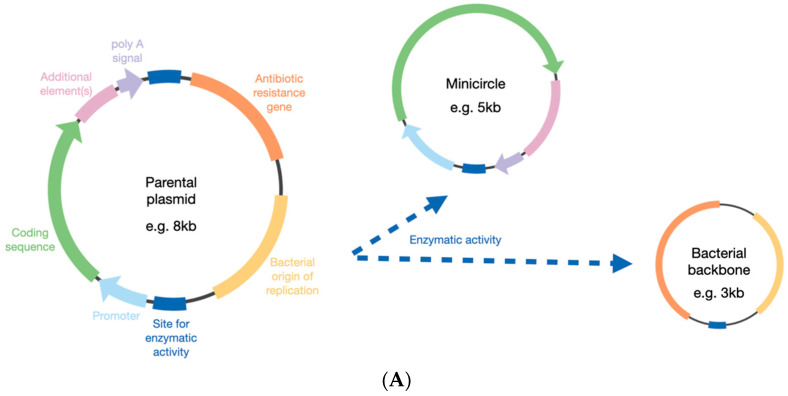

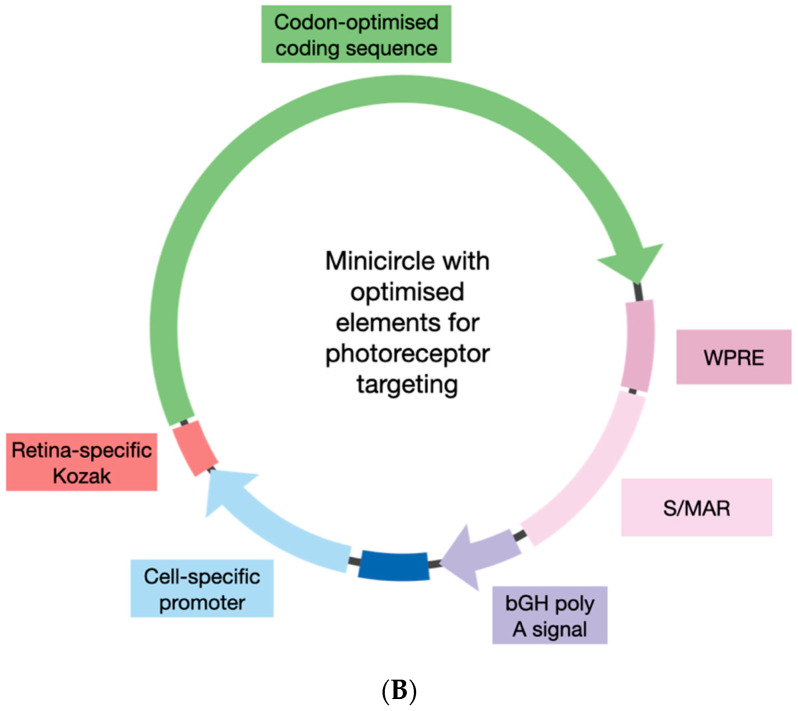
General principles in forming a minicircle from a parental plasmid. (**A**) The bacterial elements are removed from the parental plasmid by different enzymatic options (see main text). (**B**) The elements included in the minicircle can be optimised depending on the cell target, such as the photoreceptor cells. bGH = bovine growth hormone; S/MAR = scaffold matrix attachment region; WPRE = Woodchuck hepatitis virus post-transcriptional regulatory element.

DNA minicircles were first described in 1997 [6] and are generated from a parental plasmid (Figure 1). The transgene of interest is cloned into the plasmid backbone, but the bacterial elements of the plasmid are then removed, which can be achieved by the activity of enzymes such as Cre-mediated excision [7], Flp recombinase [8], λ integrase [9,10], or ϕC31 integrase [11]. Initial concerns over the production quality of minicircles have since been allayed, and yields have been improved in addition to reducing contamination from the parental plasmid [12,13]. In doing this, the size of the construct can be reduced by 2–3 kb, depending on the backbone size in the parental plasmid.

The predominant premise for minicircle use was to reduce the likelihood of transgene silencing. It was shown that expression from plasmid DNA could only be achieved for a short period of time in vivo, after which the expression rapidly dropped, despite continued plasmid survival [14]. Just as some transgene promoters are silenced via methylation of CpG dinucleotides [15], such motifs in the bacterial plasmid backbone may also induce such an effect, with CpG-depleted constructs providing improved expression profiles [16,17]. A direct comparison of different plasmid structures indicated that a greater frequency of CpG dinucleotides was associated with gene silencing [18] and transgene silencing in plasmids coincided with an increase in heterochromatin-associated histone modifications. By contrast, minicircle DNA histone modifications resembled those associated with euchromatin, and therefore remained in an active chromatin state [19,20].

The decrease in susceptibility to silencing has led to minicircles outperforming plasmid delivery in a variety of studies. For example, in mouse liver, minicircle delivery of an alpha-1 antitrypsin (AAT) expression cassette enabled higher and sustained serum AAT levels than the equivalent plasmid and linear DNA sequences [11]. Similarly, minicircle delivery to mouse heart enabled more robust and sustained luciferase reporter activity than the plasmid, which lasted for over 90 days [21]. In mouse muscle, minicircles provided 2.4–10.8 times higher green fluorescent protein (GFP) expression than equivalent plasmids and again provided more sustained expression of the reporter over time [22]. Investigations in mouse lungs yielded similar results, with minicircles achieving 6.5 times higher levels of luciferase activity compared to the equivalent plasmid-treated samples and, once more, expressions from minicircle-treated mice were sustained at a higher level and for longer than in plasmid-treated mice [23]. For ARPE19 cells, an in vitro model of the retinal pigment epithelium, minicircles also outperformed plasmid delivery when assessing GFP reporter expression from equivalent transgenes [24]. Following the delivery of these constructs to the neural retina, GFP expression was observed but not quantitatively compared [24]. Minicircle delivery to the eye will be considered further later on in this review, but it is clear from the data described so far that minicircles offer therapeutic advantages by increased and sustained transgene expression compared to plasmid vectors.

## 3. Optimising Minicircle Design

Whilst minicircles themselves are an optimised form of plasmid, further consideration of the contained DNA elements could enhance their therapeutic ability. For example, in AAV vector delivery to the neural retina, a combination of *cis*-regulatory elements within transgenes can play an important role in toxicity and efficacy [25]. Transgene optimisations for the treatment of inherited retinal disease include the use of a retinal-specific Kozak consensus for increased translational rates [26] and the inclusion of a Woodchuck hepatitis virus post-transcriptional regulatory element for transcript stability [27]. An element that has also been shown to enhance transgene expression is a scaffold/matrix attachment region (S/MAR). These sequences are AT-rich and low in CpG dinucleotides [28] and have been employed in minicircles to improve the expression profile [8]. Comparisons of plasmid and minicircle vectors (containing luciferase reporter transgenes), with and without an S/MAR, delivered in vivo to mouse liver showed that inclusion of the S/MAR in plasmid DNA was able to offset the silencing mediated by the bacterial backbone, whilst the best performing vector was the minicircle that contained an S/MAR [29]. Expressions from this construct were maintained up to 92 days post-treatment and luciferase activity at this time point was ~fivefold higher than that observed at 24 h. In retinal studies, the inclusion of an S/MAR has been indicated to improve gene expression of plasmid-delivered reporter transgenes in the retinal pigment epithelium (RPE) of wild-type mice [30]. However, mice in this study were treated as neonates, and only low levels of reporter GFP were observed, making it difficult to reliably infer improvements associated with the S/MAR. A more convincing in vivo study generated plasmids containing the vitelliform macular dystrophy 2 (*VMD2*) RPE-specific promoter, with or without an S/MAR, which were delivered by subretinal injection to adult wild-type mice, either as naked plasmid DNA or compacted as nanoparticles [31]. The inclusion of the S/MAR enabled long-term GFP expressions in the RPE (up to 120 days) with no significant differences between nanoparticle or naked plasmid DNA-treated eyes. Furthermore, the detection of GFP mRNA was comparable between 120 and 360 days post-injection, with GFP fluorescence still evident in mice two years post-injection. The researchers then swapped the GFP for the retinal pigment epithelium-specific protein 65 coding sequence (*RPE65*) in the VMD2.S/MAR expression plasmid. This was then provided by subretinal injection into *Rpe65-/-* mice, a model of retinitis pigmentosa resulting from the absence of Rpe65 in the RPE cells [32]. At 180 days post-treatment, eyes that received naked plasmid DNA or nanoparticle-compacted plasmid DNA expressed RPE65 at 50% of the level of wild-type mice. This study will be further discussed later in the review, but it is mentioned here as an example of the improvement achieved in vivo by the inclusion of an S/MAR.

Combined, these data suggest that the inclusion of an S/MAR would be worthwhile in a gene therapy minicircle. When it comes to transgene design for AAV vectors, it is necessary to minimize the elements included, as there is a packaging limitation, whereas for minicircles, there is scope to include such enhancing elements. The original S/MAR used in the described studies was derived from β-interferon and was ~2.2 kb in length, but shorter options (540–581 bp) have been assessed and proven to be as effective in vitro [33]. Other sequences to consider include those that encourage the nuclear import of the minicircle DNA, such as an SV40 enhancer [34]. Whereas AAV vectors can deliver transgenes to the nucleus efficiently, plasmid or minicircle DNA entry into post-mitotic cells, such as photoreceptors, is difficult. The inclusion of a DNA nuclear target sequence such as the NF-κB derived 3NF is a current option that has shown some indications of success in vitro and in vivo but has yet to be tested in retinal models [35].

The inclusion of these enhancer elements would increase the size of the minicircle and, whilst larger plasmids have been shown to have a negative impact on transgene expression [36,37], these are likely to be related to the CpG-silencing effects as previously discussed, which minicircles should, in theory, not encounter. There may be a currently unknown limit to how large a minicircle can be, but there is certainly more scope to encode larger transgenes than for AAV vectors. This is perhaps one of the strongest arguments for continuing to test minicircle delivery to the neural retina. Despite all the advancements in recent years, the gene supplementation of large genes for autosomal recessive disorders, such as Stargardt disease and Usher syndrome, has not progressed beyond pre-clinical studies [38,39,40,41]. Furthermore, the future of gene therapy for inherited retinal diseases will be dominated by CRISPR-based gene editing [42,43,44,45], which currently requires the delivery of large constructs, although smaller variants are being identified [46]. Although a systematic comparison of retinal delivery of minicircles of increasing size has yet to be presented, the success of larger plasmids containing an S/MAR to express transgenes in the neural retina in the long term, combined with the evidence of minicircle improvements over plasmids, suggest that minicircle size should not be an issue if it can be efficiently delivered into the target cells.

## 4. Delivery of Minicircles to the Retina

### 4.1. Electro-Transfer

For the treatment of inherited retinal disease, the primary cell types to be targeted are the photoreceptors and/or RPE cells. Whilst the RPE can perform both phagocytic and endocytic processes [47], photoreceptor cells are not able to phagocytose, and their endocytic ability is unclear. Electroporation has been developed as an innovative non-viral gene transfer treatment; it uses short high-voltage pulses to create transient pores in the plasma membrane. For in vivo applications in the retina, the electroporation conditions need to be optimised and adjusted, based on the desired direction of delivery (i.e., towards the photoreceptors or the RPE), to encourage DNA uptake whilst minimising the risk of permanent damage. The first successful retinal gene transfer by electroporation was achieved in the retinal ganglion cells [48]. Since then, many research groups have combined subretinal delivery of circular DNA vectors with electroporation. Following subretinal delivery, the electroporation of neonatal mice and rat eyes showed effective reporter expression across ~50% of the retina, including the photoreceptors [49]. More recently, the electroporation of neonate mice carrying the human rhodopsin (Rho) P23H mutation was applied to test gene editing constructs [50]. In this study, neonates received a subretinal injection of a CRISPR construct targeting the P23H mutation plus a reporter EGFP plasmid, followed by electroporation. After 7 days, editing of the Rho P23H locus was assessed, and on-target rates between 4 and 33% were achieved in GFP-positive cells. Whilst these studies provide evidence of successful delivery of circular DNA sequences to the neural retina, the structure of the retina at early developmental stages is very different to that of adult mice, which can pose a challenge for in vivo applications of electroporation in adult retinae. Other studies have combined subretinal injection and electroporation in adult mice, but the focus has been on the delivery to the RPE cells [51]. Indeed, the subretinal injection of a luciferase reporter plasmid with the RPE-specific *VMD2* promoter in adult mice (6–8 weeks of age) followed by electroporation enabled sustained luciferase expression up to 56 days post-treatment [52]. Similarly, when adult rats received subretinal or intravitreal injection of a GFP reporter plasmid followed by electroporation, GFP expression was observed in the RPE and retinal ganglion cells, respectively, but there was a distinct failure to target the photoreceptors [53]. Therefore, the delivery of minicircles to RPE in adult mice/rats should, in theory, be achievable when combined with electroporation. However, no such delivery to the photoreceptors has been convincingly demonstrated to date. It is worth noting that a clinical trial combining the injection of naked plasmid DNA into the ciliary muscle, followed by electroporation, is ongoing (NCT03308045). The Eyevensys trial is used for non-infectious uveitis, with the delivery of plasmid (pEYS606) intended to convert the ciliary muscle into a biofactory to produce a soluble anti-TNFα molecule [54]. Despite the fact that the neural retina is not targeted, this first-in-human application of electroporation to the eye for the purposes of gene therapy nevertheless paves the way for future trials.

An alternative to electroporation is iontophoresis, which uses an electrical field to permeate ionised molecules across membranes [55]. The transscleral delivery of GFP plasmid to adult mice appeared to achieve reporter expression in the photoreceptor inner and outer segments from 3 days post-iontophoresis [55]. This was observed whether a single high dose of plasmid was provided or repeat applications of a lower dose. The technique was then optimized for delivery into retinal degeneration 1 (*rd1*) pups, a mouse model in which there is a lack of photoreceptors due to an absence of functional phosphodiesterase 6B (Pde6b). A plasmid carrying a transgene of the *PDE6B* promoter and *PDE6B* coding sequence was delivered by iontophoresis. At postnatal day 23, an outer nuclear layer developed in the treated peripheral area up to 46% of the thickness of wild-type controls. These results are of interest and suggest the delivery of plasmid DNA by iontophoresis can provide a therapeutic outcome. Unfortunately, it is the only presentation of such work, with no similar studies published since then, which makes it difficult to fully assess.

### 4.2. Other Modes of Delivery

**Figure 2:**
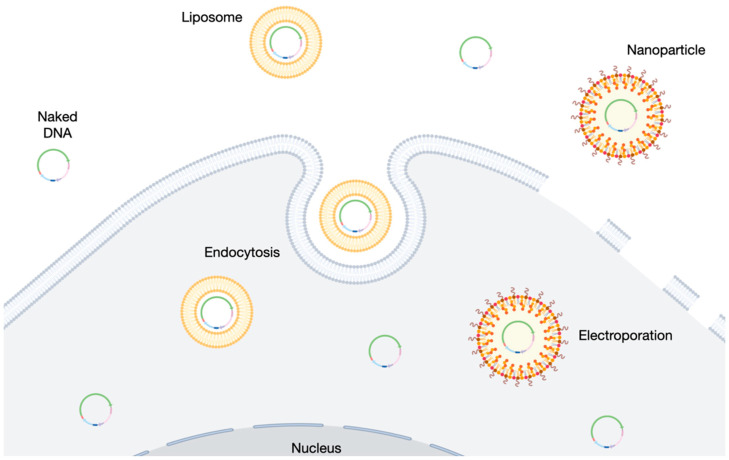
Overview of common delivery modes for minicircle entry into the target cell, including naked DNA delivery, liposome-based transfection, and electroporation.

Other methods to encourage cells in the retina to take up DNA have been attempted without the application of electro-transfer. Cationic lipid-based delivery systems can be used to complex with negatively charged DNA (forming liposomes, Figure 2) and numerous commercially available products based on this technology are available. Two such transfection reagents (Lipofectamine 2000, Thermo Fisher Scientific and NeuroPorter, Merck) were compared in the subretinal delivery of luciferase reporter plasmid in 6–8-week-old mice. Luciferase activity was observed in the RPE three days after lipofectamine–plasmid delivery with similar reporter profiles to eyes that received the same plasmid by electroporation [56]. The detection of luciferase dropped over time to become undetectable by 28 days post-treatment, with thinning of the outer nuclear layer observed. Neuroporter also enabled strong, but not uniform, staining in RPE cells that could still be seen after 4 days with no retinal thinning observed. The reduction in luciferase activity over time is not unsurprising, based on previously discussed issues relating to transgene silencing in plasmids. It is encouraging that commercially available transfection reagents enabled plasmid delivery to the retina. However, as with electroporation studies to date, it remains unclear whether the effective transfection of photoreceptors can be achieved. Direct in vivo delivery of minicircles complexed with transfection reagents have been minimally reported in the literature. Lipofectamine 2000 or cationic niosomes were prepared with minicircle DNA carrying a GFP reporter transgene and were delivered either by intravitreal or subretinal injection in adult rats [24]. Evidence of GFP expressions in the ganglion cell layer was observed after the intravitreal injection of both lipofectamine and niosome-treated eyes, whereas subretinal injections provided sporadic signs of GFP expression, primarily in the RPE and inner nuclear layer. However, no co-staining was performed, so it is difficult to identify the transfected cell types. Chloroquine-containing niosomes have also been used to deliver GFP reporter plasmid to the adult rat retina. GFP expression was observed around the site of injection 72 h after subretinal injection, including the RPE and photoreceptor cells [57].

A further example of minicircle delivery and survival in the mouse retina was presented when dissociated retinal cells from *Rho-/-GFP+* neonates were transfected with minicircles using another transfection reagent, NanoJuice (Merck) [58]. Being derived from *Rho-/-GFP*+ mice, these cells had an absence of native mouse rhodopsin, but expressed GFP in rod photoreceptors (from the neural retina leucine zipper, *Nrl,* promoter). The transfected minicircles carried a transgene for the expression of human rhodopsin and a DsRed reporter. DsRed expression was observed within 12 h of transfection, and on the third day of culture, the cells were transplanted into 3-month-old *Rho-/-GFP-* mice, which lacked photoreceptor cells due to the absence of rhodopsin. Three months post-transplantation, a layer of GFP+ cells that exhibited DsRed co-expression persisted, and rhodopsin expression was detected in ~60% of GFP-DsRed-positive cells. Whilst this study did not directly deliver the minicircles using transfection reagent to an adult retina, it revealed the long-term stability following the transfection of minicircles in photoreceptor cells.

The cationic polymer polyethylenimine (PEI) has also been used for the intravitreal delivery of plasmid DNA into the mouse eye [59]. The construct carried a transgene that enabled reporter DsRed expression in addition to a short hairpin RNA for the knockdown of melanopsin. Retinal ganglion cells were successfully transfected across a relatively large area (estimated by the authors to be ~75%). Melanopsin knockdown was confirmed in DsRed-positive cells, with an associated impact on pupil constriction (which is regulated by melanopsin) that lasted up to 60 days post-injection. However, cytotoxic effects following PEI delivery have been observed in vitro in ARPE19 cells [60], and the in vivo study provided no data on retinal structure, so it is unknown whether the PEI application incurred any toxic effects.

A particularly encouraging non-viral delivery mode that could be combined with minicircles for retinal gene therapy is polyethylene glycol (PEG)-substituted polylysine (CK30PEG) nanoparticles. The subretinal delivery of GFP reporter plasmid compacted with these nanoparticles into adult mice led to GFP expression two days post-treatment. However, GFP expression was severely downregulated by day 4, perhaps due to the previously discussed transgene silencing issues [61]. Importantly, this study found no signs of inflammatory responses induced by the subretinal injection of these nanoparticles, which have since been used in several studies and in a variety of mouse models of retinal disease. In retinal degeneration slow (*Rds*) mice, retinal degeneration occurs due to mutant peripherin, and the heterozygous model exhibits early-onset progressive rod degeneration [62,63]. A plasmid carrying a mouse rhodopsin promoter was tested for driving the expression of human peripherin, both as naked DNA and as compacted nanoparticles, and delivered by subretinal injection into *Rds+/−* mice at postnatal day 5 or 22 [64]. The delivery of pure plasmid provided no detection of peripherin expression above the baseline, whereas nanoparticle-injected eyes showed a significant increase at days 2, 7, 14, 21, 30, and 120 post-injection in mRNA transcripts extracted from neural retinae. In concordance with this, the immunostaining of peripherin was achieved in nanoparticle-treated eyes, whilst western blot analysis showed increased levels of the photoreceptor proteins (rhodopsin, peripherin, and retina outer segment membrane protein 1) in nanoparticle-treated eyes. These results were supported by improvements in photopic ERG function at postnatal day 22 that responded at levels equivalent to the wild-type controls. Following on from this success, the same nanoparticles were then used to deliver a plasmid carrying the complete *ABCA4* coding sequence into *Abca4-/-* mice. Full-length ABCA4 protein was subsequently observed by Western blot analysis of treated eyes, which peaked at 2 months post-injection and was still evident at 8 months [65]. The CK30PEG nanoparticles were again used in a study (referred to earlier in this review) for the delivery of a plasmid combining the RPE-specific *VMD2* promoter driving *RPE65* expression, including an S/MAR in the transgene [31]. *Rpe65-/-* mice were injected at postnatal day 16, and the expression of *RPE65* from the S/MAR construct was found to persist up to 15 months post-injection, yet this was observed in mice that received naked plasmid or nanoparticle-compacted DNA [66]. It is interesting that the delivery of the S/MAR-containing plasmid in this study achieved a similar success to when the plasmid was delivered by nanoparticles. Few other studies have achieved long-term success with naked plasmid delivery *in vivo*, but it is important to note that this was achieved in RPE cells, which are more capable of DNA uptake than photoreceptor cells. When it comes to circular DNA delivery to the photoreceptors, nanoparticles may offer a better route of delivery, due to their diverse set of physical and chemical properties, which can be adapted to suit different cell types. In a further study, a rhodopsin transgene was compacted to nanoparticles and delivered to *Rho-/-* mice [67]. An S/MAR element was included in the plasmid, and the delivery of the full rhodopsin genomic sequence (plasmid 10.6 kb) was compared to the delivery of the rhodopsin coding sequence only (plasmid 7 kb). The subretinal delivery of both nanoparticle-compacted plasmids showed rhodopsin expression in photoreceptors at 1 month post-injection. By 8 months post-injection, mice that received the nanoparticles with the full genomic rhodopsin sequence continued to show rhodopsin expression, whereas the cDNA-treated eyes did not. With such encouraging results across a variety of mouse models, in which the target cells were either RPE or photoreceptors, the CK30PEG nanoparticles seem a promising option for the future delivery of minicircle DNA.

## 5. Concluding Remarks

The delivery of circular DNA to the neural retina has achieved varied levels of success in the last two decades (Table 1). However, there are encouraging signs that improvements in minicircle designs could lead to them outperforming the results achieved so far with plasmid DNA. The need to deliver larger therapeutic constructs is growing as the field of gene editing expands, and minicircles offer an enticing opportunity. The issues of poor DNA uptake into the neural retina, particularly the photoreceptor cells, are slowly being overcome by improved transfection reagents, electroporation techniques, and nanoparticle development. Combining these modes of delivery with optimised minicircle constructs could provide the next leap in gene therapy for the treatment of inherited retinal diseases.

## Figures and Tables

**Table 1:** Summary of studies investigating plasmid and minicircle delivery to the eye.

Species	DNA Structure	Other Element(s)	Delivery Mode(s)	Surgical Method	Transfected Cell Types	Reference
Rats (adult)	Minicircle and plasmid		Transfection reagent (Lipofectamine 2000) and Niosomes	Intravitreal injection	Ganglion cells	[24]
	Minicircle and plasmid		Transfection reagent (Lipofectamine 2000) and Niosomes	Subretinal injection	Inner nuclear layer and RPE	[24]
Mice (pups)	Plasmid	S/MAR	Transfection reagent (FuGENE)	Intravitreal injection	Ganglion cells	[30]
Mice (adult)	Plasmid	*VMD2* promoter and S/MAR	Naked DNA and nanoparticle	Subretinal injection	RPE	[31]
*Rpe65-/-* mice (adult)	Plasmid	*VMD2* promoter and S/MAR	Naked DNA and nanoparticle	Subretinal injection	RPE	[31,66]
Mice and rats (pups)	Plasmid		Electroporation	Subretinal injection	All retinal layers	[49]
RHO P23H mice (pups)	Plasmid		Electroporation	Subretinal injection	Photoreceptors	[50]
Mice (adult)	Plasmid	*VMD2* promoter	Electroporation	Subretinal injection	RPE	[51]
Mice (adult)	Plasmid	*VMD2* promoter	Electroporation	Subretinal injection	RPE	[52]
Rats (adult)	Plasmid		Electroporation	Intravitreal injection	Ganglion cells	[53]
	Plasmid		Electroporation	Subretinal injection	RPE	[53]
Mice (adult)	Plasmid		Iontophoresis		Photoreceptors	[55]
*rd1* mice (pups)	Plasmid	*PDE6B* promoter	Iontophoresis		Photoreceptors	[55]
Mice (adult)	Plasmid		Transfection reagent (Lipofectamine 2000 and NeuroPorter)	Subretinal injection	RPE	[56]
Rats (adult)	Plasmid		Chloroquine-containing niosomes	Subretinal injection	Photoreceptor and RPE cells	[57]
Mice (adult)	Plasmid		Transfection reagent (PEI)	Intravitreal injection	Ganglion cells	[59]
Mice (adult)	Plasmid		CK30PEG nanoparticles	Subretinal injection	Photoreceptors	[61]
*rds* mice (pups and adult)	Plasmid	*RHO* promoter	Naked DNA and CK30PEG nanoparticles	Subretinal injection	Photoreceptors	[64]
*Abca4-/-* mice (adult)	Plasmid	*IRBP* or *MOP* promoter	Naked DNA and CK30PEG nanoparticles	Subretinal injection	Photoreceptors	[65]
*Rho-/-* mice (pups)	Plasmid	*MOP* promoter and S/MAR	CK30PEG nanoparticles	Subretinal injection	Photoreceptors	[67]

## Abbreviations

AAT	alpha-1 antitrypsin
ABCA4	ATP-binding cassette subfamily A member 4
AAV	adeno-associated virus
CRISPR	clustered regularly interspersed short palindromic repeats
DsRed	red fluorescent protein derived from Discosoma coral
EGFP	enhanced green fluorescent protein
ERG	electroretinogram
GFP	green fluorescent protein
IRBP	interphotoreceptor retinoid-binding protein
MOP	mouse opsin promoter
NF-κB	nuclear factor kappa B
Nrl	neural retina leucine zipper
Pde6b	phosphodiesterase 6B
PEI	polyethylenimine
rd1	retinal degeneration 1
rds	retinal degeneration slow
Rho	rhodopsin
RPE	retinal pigment epithelium
RPE65	retinal pigment epithelium-specific protein 65
S/MAR	scaffold matrix attachment region
TNFα	tumour necrosis factor alpha
VMD2	vitelliform macular dystrophy 2
WPRE	Woodchuck hepatitis virus post-transcriptional regulatory element
